# Workaholism and quality of life: an integrative literature review

**DOI:** 10.47626/1679-4435-2021-640

**Published:** 2021-12-30

**Authors:** Taísa Vedoato, Danielli Rafaeli Candido Pedro, Izabela Melo Garcia, Maria José Quina Galdino, Patricia Aroni, Maria do Carmo Fernandez Lourenço Haddad

**Affiliations:** 1 Programa de Pós-graduação em Enfermagem, Universidade Estadual de Londrina (UEL), Londrina, PR, Brazil.; 2 Departamento de Enfermagem, Universidade Estadual do Norte do Paraná, Bandeirantes, PR, Brazil.; 3 Departamento de Enfermagem, UEL, Londrina, PR, Brazil.

**Keywords:** quality of life, working conditions, occupational health, occupational diseases, work, qualidade de vida, condições de trabalho, saúde do trabalhador, doenças profissionais, trabalho

## Abstract

This study aimed to analyze the scientific evidence available in the literature that addresses the relationship between workaholism-workaholic and quality of life. This was an integrative review, whose search was conducted on the following databases: MEDLINE, Latin American and Caribbean Literature in Health Sciences, Web of Science, and Scopus. The survey found 10 primary articles published from 2011 to 2018, of which eight were published in English and two in Portuguese. Only 20% of the investigations included health care professionals in the hospital setting, of which one was conducted in Korea and the other in Brazil. It was concluded that workaholism was associated with workers’ quality of life in all studies, consolidating the fact that the workaholism phenomenon has negative consequences to workers’ quality of life.

## Resumo

Este estudo teve como objetivo analisar as evidências científicas disponíveis na literatura que abordem a relação *workaholism-workaholic* com qualidade de vida. Tratou-se de uma revisão integrativa, por meio de consulta às bases de dados MEDLINE, Literatura Latino-Americana e do Caribe em Ciências da Saúde, Web of Science e Scopus. Encontraram-se 10 artigos primários publicados entre 2011 e 2018, dos quais oito foram publicados em inglês e dois, em português. Apenas 20% das pesquisas incluídas foram conduzidas com trabalhadores de saúde no ambiente hospitalar, sendo uma delas na Coreia e a outra, no Brasil. Conclui-se que o *workaholism* associou-se à qualidade de vida dos trabalhadores em todos os estudos, consolidando que o fenômeno *workaholism* acarreta consequências negativas à qualidade de vida dos trabalhadores.

## INTRODUCTION

Workaholism is typically described as a chronic pattern of high work investment, long working hours, working beyond organization expectations, and an uncontrollable obsession with work. Although the concept has been associated with positive attributes, such as extraordinary work effort, most scholars currently regard workaholism mainly as a negative entity, because it is associated with health problems, low job and life satisfaction, work-family conflicts, and sleep problems, as well as impaired job performance.^1^

The term workaholism is characterized by the combination of two dimensions: a behavioral one, characterized by working excessively, i.e., when an individual invests too much time and energy in work, much beyond the expected; and a cognitive one, characterized by working compulsively due to an irresistible drive to be involved in work-related matters.^2^

The main characteristics of workaholic individuals are being intensively absorbed with work, with long working hours, excessive workload, fast work pace, and unrestrained desire for results. These factors lead to a distortion in the individual- organization relationship and impair people’s quality of life, resulting in serious health consequences.^3^

Physically, workaholism is manifested by extreme tiredness, hypertension, insomnia, gastritis, alopecia, and vascular and heart problems. However, since these symptoms alone did not express a possible work addiction, a supplementary diagnosis should also analyze worker’s psychological and social aspects. A lifestyle based on workaholism can effectively lead to high levels of anxiety and depression, having a negative impact on quality of life.^4^

The World Health Organization (WHO) Quality of Life Group conceptualized quality of life as the individuals’ perception of their own life condition, within their own context of culture and value system, considering their life goals, expectations, and concerns. Health-related quality of life refers to individuals’ perception on their life condition in the face of the disease and the consequences and treatments related to it, i.e., how the disease affects their life condition. Measuring this perception is very subjective, because of individuals’ difficulty in relating their dysfunction to the multiple dimensions of their life.^5^

Poor quality of life in the workplace may have negative consequences, such as increased absenteeism, reduced productivity, lack of interest in work activities, increased number of occupational accidents, apathy, muscular tension, tachycardia, headache, depression, and sleep changes, in addition to other physical, psychological, and social strains. These impacts, in turn, may interfere with individual’s willingness to perform their work activities.^6^

In view of the foregoing, this study is justified by the need to compile the studies that addressed the relationship between workaholism-workaholic and quality of life so as to improve scientific knowledge on the theme and help mangers understand the dynamics of workaholic professionals and the characteristics that may impair their quality of life. This integrative review aimed to analyze the scientific evidence available in the literature that addresses the relationship between workaholism-workaholic with quality of life.

## METHODS

This was an integrative review, a method that gathers, assesses, and synthesizes the results of investigations on a specific subject. For the development of this study, a research protocol was designed to address the methodological stages to be followed: formulation of the research question, eligibility criteria for primary studies, definition of search strategy and selection of primary articles, data extraction, assessment of the primary studies included, interpretation of results, and presentation of the review.^7^

For the formulation of the research question, we used the population, concept, and context (PCC) strategy. The use of this strategy enables to formulate the research question that guides the implementation of the review methods to identify keywords and descriptors, which help find relevant primary studies on databases.^8^ Therefore, the research question delimited for this study was the following: what scientific evidence available in the literature assesses the association between workaholism-workaholic and workers’ quality of life?

The search for primary studies was conducted in March 2019 on the following databases: MEDLINE (via PubMed), Latin American and Caribbean Health Sciences Literature (Literatura Latino-Americana e do Caribe em Ciências da Saúde, LILACS), Web of Science (WoS), and Scopus. The authors decided to include in this study articles retrieved from these databases, with no manual search of articles.

The controlled descriptors selected in the Descriptors of Health Science (Descritores em Ciências da Saúde, DeCS) of the Virtual Health Library (VHL) were quality of life, occupational health, and occupational diseases. These descriptors were used for the search on LILACS together with the following keywords: workaholic, workaholism, health-related quality of life, occupational health, occupational diseases, and occupational illnesses. Thus, the search strategy used in LILACS differed from that used in the other databases, consisting of the following strategy: ((Workaholism) OR (Workaholic)) AND ((Quality of Life) OR (Occupational Health) OR (Occupational Diseases) OR (Health-related Quality of Life) OR (Occupational Health) OR (Occupational Diseases) OR (Occupational Illnesses)).

The descriptors selected in the Medical Subject Headings (MeSH) database were quality of life, occupational health, working conditions. These descriptors were used for search on the WoS, MEDLINE, and Scopus databases, together with the following keywords: workaholic, workaholism. The search strategy was used as follows: (“workaholism” OR “workaholic”) AND (“quality of life” OR “Occupational Health” OR “Working Conditions”).

We decided to use the following keywords: “occupational diseases”, “occupational illnesses” and the descriptor “work conditions” in order to retrieve more descriptors for quality of life, which allows for a wide search and made it possible to assess the eligibility of many studies. This procedure was adopted because several studies employed these descriptors and keywords in their manuscript.

Inclusion criteria established for primary studies were articles addressing workaholism-workaholic and quality of life and written in English, Portuguese, and Spanish. Systematic and integrative review studies, response letters, editorials, and secondary studies were excluded from this study sample. The selection and analysis of primary studies were independently conducted by two reviewers. Discrepancies in article selection were solved by a third reviewer.

For the phase of data extraction, the authors themselves designed a spreadsheet containing items related to the following features: identification of article, type of study, research level of evidence, objectives, and main results.

For level of evidence, the definition of the type of study was maintained according to the authors of the investigations included in the sample. This study employed concepts proposed by nursing scholars who recommend a certain hierarchy of evidence for study analises.^9^

The level of evidence was classified into seven levels, namely: level 1, the strongest one, evidence from systematic review or meta-analysis of randomized clinical trials; level 2, evidence from well-designed randomized clinical trials; level 3, evidence from well-designed non-randomized clinical trials; level 4, evidence from well-designed cohort and case-control studies; level 5, evidences from systematic review of descriptive and qualitative studies; level 6, evidence from a single descriptive or qualitative study; and level 7, the weakest one, evidence from experts’ opinions.^9^

The analysis of the results obtained was performed in a descriptive manner, with the synthesis of each study included in the integrative review, as well as comparisons between studies, highlighting differences and similarities.

## RESULTS

In the first analysis, after reading of title and abstract of primary studies (n = 196), the articles that did not describe the association between workaholism-workaholic and quality of life were excluded (n = 177). Among them, secondary studies (n = 28) and those that did not answer the research question of the present study (n = 149) were also excluded.

**Figure 1:**
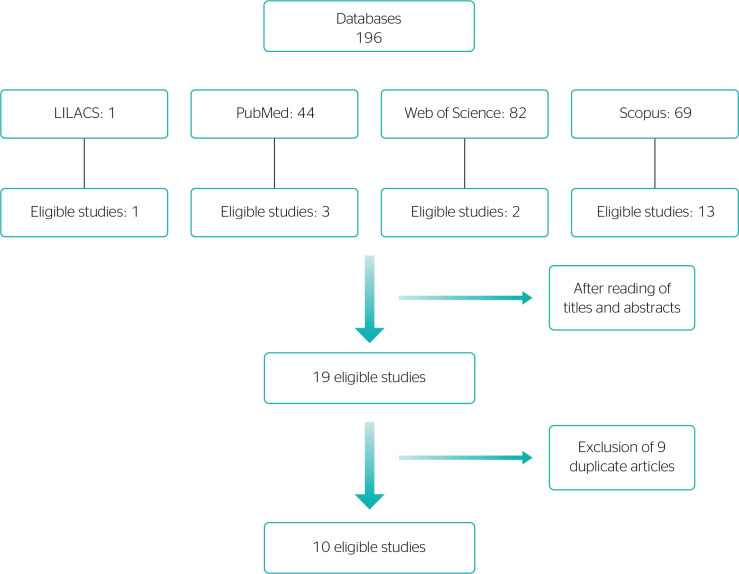
Flowchart of selection of the studies included in the integrative review on workaholic-workaholism and quality of life according to databases.

In the second analysis, articles were read in full (n = 19), and nine duplicate articles were excluded. The flowchart of study search and selection is presented in Figure 1.

This integrative review included 10 primary studies published from 2011 to 2018. With regard to their language, eight were published in English,^10-17^ and two in Portuguese.^18,19^ In relation to the method adopted in the studies, eight (80%)^10,12,13,15-19^ were cross-sectional studies, and two (20%)^11,14^ were prospective longitudinal studies.

Only 20% of the included investigations were conducted with health care professionals in the hospital setting, one of them with 278 Korean nurses in a university hospital,^12^ and the other was developed in Brazil, in the state of Paraíba, with 1,110 physicians.^19^

Two studies were conducted in Japan, one of them with 3,899 production workers^15^ and the other with 1,967 workers from different areas;^11^ two studies were performed in Brazil,^18,19^ three studies were performed in Japan,^13,15^ and the other studies were conducted in Norway,^17^ Korea,^12^ France,^16^ Italy,^14^ and Poland^10^, one study for each country.

Table 1 presents the main information obtained from the primary studies included in this integrative review.

**Table 1:** Synthesis of the primary studies included in the integrative review on workaholic-workaholism and quality of life

Year/country Database and reference Study design Level of evidence (LE)	Objectives of the study	No. of participants and main results related to workaholism and quality of life
2011/Brazil LILACS^18^ Cross-sectional LE: 6	To identify sociodemographic, labor, and psychosocial risk factors of workaholism in a sample of multifunctional workers in Porto Alegre and metropolitan area, state of Rio Grande do Sul, Brazil.	No. of participants: 471 workers who performed their activities in work organizations. The variable sex showed a significant difference, with women having higher rates of excessive work. There was a positive association with labor variables, between excessive work and contractual working hours; and between excessive work and working hours that were effectively carried out. There was a negative association between excessive work and the perception of being healthy.
2014/Poland Scopus^10^ Cross-sectional LE: 6	To verify empirically a conception of workaholism as a multidimensional syndrome.	No. of participants: 137 managers who had graduated with, or were studying to attain, a Master’s degree in Business Administration. Workaholism has a three-dimensional structure that includes behavioral, cognitive, and affective dimensions. Workaholics were less satisfied with the following aspects of quality of life: satisfaction with life, with self-achievement, and with life situation.
2013/Japan PubMed^15^ Cross-sectional LE: 6	To examine the association of workaholism with psychologically ill health, low back pain, and disability to work among Japanese workers.	No. of participants: 3,899 production workers. The groups with middle and high workaholism had significantly higher levels of depressive mood and low back pain, with a negative impact on working performance.
2018/Korea Scopus^12^ Cross-sectional LE: 6	To delineate the relationships between work addiction and professional quality of life among nurses in university hospitals.	No. of participants: 278 Korean nurses. Of participants, 46.5% had mild or high work addiction, and those with high work addiction tend to develop higher burnout.
2016/Japan Scopus^13^ Cross-sectional LE: 6	To conduct a predictive study of the pre-existing variables for workaholism.	No. of participants: 513 workers. Personality variables such as engagement, self-efficacy, obsessive-compulsive component, satisfaction with life, and lifestyle were predictive of workaholism.
2013/Italy Scopus^14^ Longitudinal LE: 6	To test a theoretical model in which workaholism predicts both directly and indirectly, via psychophysical strain, job performance and sickness absences.	No. of participants: 322 workers in a private company. Positive relationship between workaholism and psychophysics (relationship between physical stimuli and their respective sensations). Psychophysical strain was negatively associated with job performance and positively associated with sickness absences.
2013/France Scopus^16^ Descriptive LE: 6	To describe the typologies of workaholism, its mode of evolution, its diagnostic approach, the multiple negative consequences for both patient and family, as well as the principles of management based on cognitive-behavioral therapy of this disorder, which may be considered as a true addiction.	Workaholism belongs to the behavioral addictions. The differential diagnosis should distinguish a hard worker from a workaholic, who is prisoner of this compulsive behavior.
2012/Japan Scopus^11^ Prospective longitudinal LE: 6	To investigate the distinctiveness between workaholism and work engagement.	No. of participants: 1,967 workers of different companies. Workaholism was related to increased lack of health and reduced quality of life. Work engagement was related to fewer health problems and increased life satisfaction and job performance.
2011/Norway Scopus^17^ Cross-sectional LE: 6	To examine workaholism components (work involvement, drive, enjoyment of work) and potential outcomes in terms of psychological well-being and health.	No. of participants: 661 employees from six different organizations. Work enjoyment was positively associated with life satisfaction and negatively associated with symptoms of poor health. Work involvement and drive were positively related to symptoms of poor health.
2017/Brazil Web of Science^19^ Exploratory, descriptive, and cross-sectional LE: 6	To evaluate the quality of life of physicians and to investigate to what extent it is affected by work addiction.	No. of participants: 1,110 physicians. Most participants presented high quality of life. Women had lower quality of life than men. Quality of life was negatively correlated with number of shifts (p < 0.005), and the higher the addiction to work, the lower the quality of life.

LILACS: Latin American and Caribbean Literature in Health Sciences (Literatura Latino-Americana e do Caribe em Ciências da Saúde).

## DISCUSSION

Most studies highlighted the compulsive dimension of workaholism as the responsible for decreased quality of life. The individual characteristics that predict the compulsive aspect of the workaholism phenomenon include neuroticism, perfectionism, absorption, self-efficacy, whereas the variables life satisfaction, emotional stability, dedication, and nutrition were inversely associated with the phenomenon. With regard to personality variables, emotional stability was inversely related to compulsive work.^16-19^

The excessive dimension was also assessed in the studies, using the effort-reward imbalance model. There was a relationship between this dimension and work demand, suggesting that the effects of occupational stressors for health may be partially or totally mediated by workaholics.^13^

With regard to individual aspects, it is worth emphasizing that satisfaction with life is negatively related to working excessively. Conversely, organizations also have aspects that favor excessive work, such as use of information and communication technologies to continue working beyond office hours.^18^

In this same context, one of the studies^11^ compared the difference between work engagement and workaholism in terms of quality of life and job performance, reinforcing the idea that work engagement is associated with positive aspects - better quality of life and job performance -, whereas workaholism is associated with negative aspects - poor quality of life and low job performance.

Two studies^18,19^ revealed that the variable sex showed a significant difference, with women having higher levels of excessive work. A risk profile was found, consisting of female professionals with long working hours and long overtime hours, who perceive themselves as less healthy and have lower overall life satisfaction.

It bears noting that concern, understanding, and prevention of workaholism are necessary, because work addiction may affect the quality of life of nursing profissionals.^12^ Health care professionals who are work addicts eventually forget the social, physical, and mental domains of life, which are equally important for health maintenance, thus hampering their quality of life.^19^

Studies conducted in Japan and France^15,16^ confirm that work addiction may also be grouped into physiological and psychological addictions, in line with another study^4^ that stated that individuals with workaholism may also present with physical symptoms such as extreme tiredness, arterial hypertension, insomnia, gastritis, alopecia, and cardiovascular problems.

It was possible that the greater the work addiction, the lower worker’s quality of life.^20^

A limitation of this study is the fact that the 10 retrieved studies assessed different populations of workaholic workers and their relationship with quality of life, and all included studies were classified with level of evidence 6. It bears highlighting that cross-sectional studies do not enable to determine a causal relationship between workaholism and poor quality of life.

Therefore, new longitudinal investigations are still necessary, which should be conducted with objective measures that may generate studies with higher levels of evidence, so as to consolidate the relationship between workaholism and quality of life. It was also noticed that the included investigations were predominantly conducted with teams that work in production services, whereas there was a scarcity of studies with professional groups whose activities require physical and mental exertion in the work process into which they are inserted, such as teachers and health care professionals.

It was observed that, the greater the knowledge on negative events, the greater the possibility of taking actions to prevent them in advance, changing the conditions that facilitate the development of workaholism in an individual or group, which consequently will have a negative impact on worker’s productivity, mental health, and quality of life.

This research enabled to elucidate the implications of workaholism on quality of life, but additional studies are necessary to assess strategies that improve workers’ health and quality of life. We believe that the present study has shown the relevance of proposing strategies to improve workers’ health and quality of life and, consequently, their productivity and drive to work.

## CONCLUSIONS

Workaholism was associated with workers’ quality of life in all studies, which contributed to consolidate the fact that the workaholism phenomenon, regardless of the affected population, has negative consequences to workers’ quality of life. Most studies highlighted the need for contracting organizations to identify workaholic individuals and their predictive characteristics in order to guide and help workers to focus on their personal attitudes towards work.

In view of the findings, it was observed that workaholism not only compromises workers’ sociability with the work team, their family, and their health but also reduces productivity and increases the rates of absenteeism and sickness allowance, impairing the quality of the services provided. Therefore, strategies to improve quality of life in companies are necessary and may benefit workers.
